# Supplementary material for: Pyruvate Dehydrogenase Kinase 1 inhibition mediated oxidative phosphorylation enhancement in cartilage promotes osteoarthritis progression
Source: BMC Musculoskelet Disord. 2023 Jul 20;24:597. doi: 10.1186/s12891-023-06585-6 (PMC10357736; doi:10.1186/s12891-023-06585-6)
Supplement: Supplementary file 1 — Additional file 1. [file 12891_2023_6585_MOESM1_ESM.zip › BP.Gsea.1653623667859/GOBP_RIBOSOMAL_LARGE_SUBUNIT_BIOGENESIS.html]

Details for gene set GOBP\_RIBOSOMAL\_LARGE\_SUBUNIT\_BIOGENESIS[GSEA]

|  || Dataset | 55457\_collapsed\_to\_symbols.cls.cls #OA\_versus\_normal.cls.cls #OA\_versus\_normal\_repos |
| Phenotype | cls.cls#OA\_versus\_normal\_repos |
| Upregulated in class | normal |
| GeneSet | GOBP\_RIBOSOMAL\_LARGE\_SUBUNIT\_BIOGENESIS |
| Enrichment Score (ES) | -0.5054064 |
| Normalized Enrichment Score (NES) | -2.0139353 |
| Nominal p-value | 0.0 |
| FDR q-value | 0.028219678 |
| FWER p-Value | 0.227 |
Table: GSEA Results Summary

  

Fig 1: Enrichment plot: GOBP\_RIBOSOMAL\_LARGE\_SUBUNIT\_BIOGENESIS      
 Profile of the Running ES Score & Positions of GeneSet Members on the Rank Ordered List

  

| SYMBOL | TITLE | RANK IN GENE LIST | RANK METRIC SCORE | RUNNING ES | CORE ENRICHMENT || 1 | NVL | nuclear VCP like [Source:HGNC Symbol;Acc:HGNC:8070] | 73 | 0.956 | 0.0378 | No |
| 2 | RPF1 | ribosome production factor 1 homolog [Source:HGNC Symbol;Acc:HGNC:30350] | 1061 | 0.504 | -0.0161 | No |
| 3 | NSA2 | NSA2 ribosome biogenesis factor [Source:HGNC Symbol;Acc:HGNC:30728] | 1508 | 0.428 | -0.0313 | No |
| 4 | ZNHIT3 | zinc finger HIT-type containing 3 [Source:HGNC Symbol;Acc:HGNC:12309] | 1648 | 0.407 | -0.0236 | No |
| 5 | NHP2 | NHP2 ribonucleoprotein [Source:HGNC Symbol;Acc:HGNC:14377] | 2026 | 0.358 | -0.0367 | No |
| 6 | GTF3A | general transcription factor IIIA [Source:HGNC Symbol;Acc:HGNC:4662] | 2151 | 0.344 | -0.0307 | No |
| 7 | SNU13 | small nuclear ribonucleoprotein 13 [Source:HGNC Symbol;Acc:HGNC:7819] | 3795 | 0.178 | -0.1505 | No |
| 8 | SDAD1 | SDA1 domain containing 1 [Source:HGNC Symbol;Acc:HGNC:25537] | 4229 | 0.134 | -0.1782 | No |
| 9 | RPL26L1 | ribosomal protein L26 like 1 [Source:HGNC Symbol;Acc:HGNC:17050] | 4584 | 0.106 | -0.2009 | No |
| 10 | DDX28 | DEAD-box helicase 28 [Source:HGNC Symbol;Acc:HGNC:17330] | 4602 | 0.105 | -0.1975 | No |
| 11 | RPL10A | ribosomal protein L10a [Source:HGNC Symbol;Acc:HGNC:10299] | 4974 | 0.074 | -0.2230 | No |
| 12 | MRPL20 | mitochondrial ribosomal protein L20 [Source:HGNC Symbol;Acc:HGNC:14478] | 5989 | -0.004 | -0.3018 | No |
| 13 | RSL24D1 | ribosomal L24 domain containing 1 [Source:HGNC Symbol;Acc:HGNC:18479] | 5992 | -0.004 | -0.3018 | No |
| 14 | EIF6 | eukaryotic translation initiation factor 6 [Source:HGNC Symbol;Acc:HGNC:6159] | 6140 | -0.015 | -0.3125 | No |
| 15 | NOP53 | NOP53 ribosome biogenesis factor [Source:HGNC Symbol;Acc:HGNC:4333] | 6334 | -0.031 | -0.3261 | No |
| 16 | RPL35A | ribosomal protein L35a [Source:HGNC Symbol;Acc:HGNC:10345] | 6383 | -0.034 | -0.3283 | No |
| 17 | RPL10L | ribosomal protein L10 like [Source:HGNC Symbol;Acc:HGNC:17976] | 6502 | -0.042 | -0.3356 | No |
| 18 | NLE1 | notchless homolog 1 [Source:HGNC Symbol;Acc:HGNC:19889] | 7253 | -0.099 | -0.3895 | No |
| 19 | RPL35 | ribosomal protein L35 [Source:HGNC Symbol;Acc:HGNC:10344] | 7563 | -0.124 | -0.4079 | No |
| 20 | FASTKD2 | FAST kinase domains 2 [Source:HGNC Symbol;Acc:HGNC:29160] | 7730 | -0.136 | -0.4146 | No |
| 21 | DDX18 | DEAD-box helicase 18 [Source:HGNC Symbol;Acc:HGNC:2741] | 7996 | -0.154 | -0.4282 | No |
| 22 | RPL3L | ribosomal protein L3 like [Source:HGNC Symbol;Acc:HGNC:10351] | 8097 | -0.163 | -0.4286 | No |
| 23 | MAK16 | MAK16 homolog [Source:HGNC Symbol;Acc:HGNC:13703] | 8098 | -0.163 | -0.4212 | No |
| 24 | DHX30 | DExH-box helicase 30 [Source:HGNC Symbol;Acc:HGNC:16716] | 8111 | -0.164 | -0.4147 | No |
| 25 | RSL1D1 | ribosomal L1 domain containing 1 [Source:HGNC Symbol;Acc:HGNC:24534] | 8286 | -0.177 | -0.4201 | No |
| 26 | WDR12 | WD repeat domain 12 [Source:HGNC Symbol;Acc:HGNC:14098] | 8505 | -0.194 | -0.4283 | No |
| 27 | EBNA1BP2 | EBNA1 binding protein 2 [Source:HGNC Symbol;Acc:HGNC:15531] | 8931 | -0.226 | -0.4511 | No |
| 28 | FTSJ3 | FtsJ RNA 2'-O-methyltransferase 3 [Source:HGNC Symbol;Acc:HGNC:17136] | 9193 | -0.247 | -0.4602 | No |
| 29 | RRP15 | ribosomal RNA processing 15 homolog [Source:HGNC Symbol;Acc:HGNC:24255] | 9775 | -0.299 | -0.4918 | Yes |
| 30 | WDR74 | WD repeat domain 74 [Source:HGNC Symbol;Acc:HGNC:25529] | 9777 | -0.299 | -0.4783 | Yes |
| 31 | URB1 | URB1 ribosome biogenesis homolog [Source:HGNC Symbol;Acc:HGNC:17344] | 9833 | -0.304 | -0.4687 | Yes |
| 32 | NIP7 | nucleolar pre-rRNA processing protein NIP7 [Source:HGNC Symbol;Acc:HGNC:24328] | 9950 | -0.316 | -0.4633 | Yes |
| 33 | RPL5 | ribosomal protein L5 [Source:HGNC Symbol;Acc:HGNC:10360] | 10201 | -0.341 | -0.4673 | Yes |
| 34 | ZNHIT6 | zinc finger HIT-type containing 6 [Source:HGNC Symbol;Acc:HGNC:26089] | 10220 | -0.343 | -0.4531 | Yes |
| 35 | RPL24 | ribosomal protein L24 [Source:HGNC Symbol;Acc:HGNC:10325] | 10263 | -0.347 | -0.4405 | Yes |
| 36 | PES1 | pescadillo ribosomal biogenesis factor 1 [Source:HGNC Symbol;Acc:HGNC:8848] | 10268 | -0.347 | -0.4250 | Yes |
| 37 | BRIX1 | biogenesis of ribosomes BRX1 [Source:HGNC Symbol;Acc:HGNC:24170] | 10275 | -0.348 | -0.4096 | Yes |
| 38 | MRTO4 | "MRT4 homolog, ribosome maturation factor [Source:HGNC Symbol;Acc:HGNC:18477]" | 10300 | -0.350 | -0.3955 | Yes |
| 39 | NOL9 | nucleolar protein 9 [Source:HGNC Symbol;Acc:HGNC:26265] | 10361 | -0.357 | -0.3840 | Yes |
| 40 | RPL10 | ribosomal protein L10 [Source:HGNC Symbol;Acc:HGNC:10298] | 10684 | -0.391 | -0.3912 | Yes |
| 41 | RPL7A | ribosomal protein L7a [Source:HGNC Symbol;Acc:HGNC:10364] | 10811 | -0.406 | -0.3825 | Yes |
| 42 | NOP16 | NOP16 nucleolar protein [Source:HGNC Symbol;Acc:HGNC:26934] | 10959 | -0.425 | -0.3746 | Yes |
| 43 | RRS1 | ribosome biogenesis regulator 1 homolog [Source:HGNC Symbol;Acc:HGNC:17083] | 11045 | -0.437 | -0.3614 | Yes |
| 44 | RPL38 | ribosomal protein L38 [Source:HGNC Symbol;Acc:HGNC:10349] | 11122 | -0.446 | -0.3470 | Yes |
| 45 | RPL6 | ribosomal protein L6 [Source:HGNC Symbol;Acc:HGNC:10362] | 11129 | -0.447 | -0.3271 | Yes |
| 46 | RPL14 | ribosomal protein L14 [Source:HGNC Symbol;Acc:HGNC:10305] | 11154 | -0.451 | -0.3084 | Yes |
| 47 | RPL11 | ribosomal protein L11 [Source:HGNC Symbol;Acc:HGNC:10301] | 11252 | -0.464 | -0.2948 | Yes |
| 48 | PAK1IP1 | PAK1 interacting protein 1 [Source:HGNC Symbol;Acc:HGNC:20882] | 11345 | -0.478 | -0.2802 | Yes |
| 49 | LAS1L | LAS1 like ribosome biogenesis factor [Source:HGNC Symbol;Acc:HGNC:25726] | 11474 | -0.498 | -0.2675 | Yes |
| 50 | GTPBP4 | GTP binding protein 4 [Source:HGNC Symbol;Acc:HGNC:21535] | 11861 | -0.562 | -0.2720 | Yes |
| 51 | HEATR3 | HEAT repeat containing 3 [Source:HGNC Symbol;Acc:HGNC:26087] | 11901 | -0.572 | -0.2490 | Yes |
| 52 | MDN1 | midasin AAA ATPase 1 [Source:HGNC Symbol;Acc:HGNC:18302] | 11973 | -0.588 | -0.2277 | Yes |
| 53 | PPAN | peter pan homolog [Source:HGNC Symbol;Acc:HGNC:9227] | 12022 | -0.597 | -0.2043 | Yes |
| 54 | NOC2L | NOC2 like nucleolar associated transcriptional repressor [Source:HGNC Symbol;Acc:HGNC:24517] | 12258 | -0.663 | -0.1924 | Yes |
| 55 | RPLP0 | ribosomal protein lateral stalk subunit P0 [Source:HGNC Symbol;Acc:HGNC:10371] | 12285 | -0.672 | -0.1638 | Yes |
| 56 | RPL3 | ribosomal protein L3 [Source:HGNC Symbol;Acc:HGNC:10332] | 12350 | -0.691 | -0.1373 | Yes |
| 57 | NOP2 | NOP2 nucleolar protein [Source:HGNC Symbol;Acc:HGNC:7867] | 12372 | -0.699 | -0.1071 | Yes |
| 58 | RPL7 | ribosomal protein L7 [Source:HGNC Symbol;Acc:HGNC:10363] | 12519 | -0.757 | -0.0840 | Yes |
| 59 | NPM1 | nucleophosmin 1 [Source:HGNC Symbol;Acc:HGNC:7910] | 12530 | -0.762 | -0.0501 | Yes |
| 60 | BOP1 | BOP1 ribosomal biogenesis factor [Source:HGNC Symbol;Acc:HGNC:15519] | 12580 | -0.785 | -0.0182 | Yes |
| 61 | RPL23A | ribosomal protein L23a [Source:HGNC Symbol;Acc:HGNC:10317] | 12785 | -0.944 | 0.0090 | Yes |
Table: GSEA details [plain text format]

  

Fig 2: GOBP\_RIBOSOMAL\_LARGE\_SUBUNIT\_BIOGENESIS      
 Blue-Pink O' Gram in the Space of the Analyzed GeneSet

  

Fig 3: GOBP\_RIBOSOMAL\_LARGE\_SUBUNIT\_BIOGENESIS: Random ES distribution      
 Gene set null distribution of ES for **GOBP\_RIBOSOMAL\_LARGE\_SUBUNIT\_BIOGENESIS**
